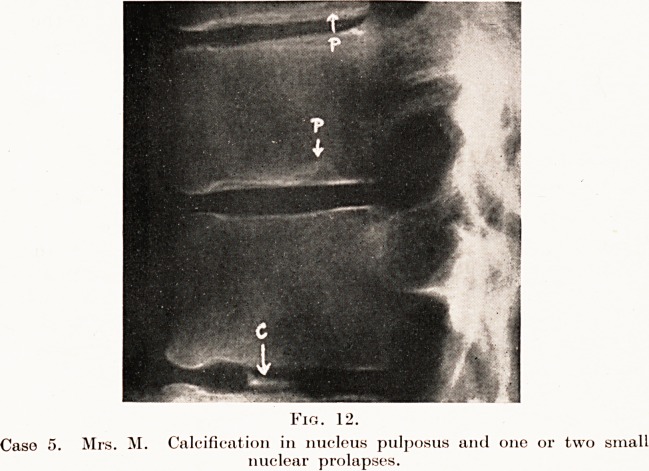# The Clinical Importance of the Intervertebral Discs, with Special Reference to Nuclear Prolapses

**Published:** 1934

**Authors:** Gilbert B. Bush

**Affiliations:** Hon. Assistant Radiologist, Bristol General Hospital; Hon. Radiologist, Weston-super-Mare Hospital


### THE CLINICAL IMPORTANCE OF THE INTERVERTEBRAL DISCS, WITH SPECIAL REFERENCE TO NUCLEAR PROLAPSES.

BY

GILBERT B. BUSH, M.B., Ch.B., D.M.R.E.,

*Hon. Assistant Radiologist, Bristol General Hospital ;*
*Hon. Radiologist, Weston-super-Mare Hospital.*

---

THE purpose of this communication is to draw your attention to the importance of the intervertebral discs in the structure and function of the spine, and to illustrate my remarks by reference to a condition found mainly in young adults, the pathology of which has been somewhat obscure until within the last year or two, and which is characterized by a mild degree of kyphosis which may be progressive, together with a varying amount of pain in the back. The condition is clinically important in that, by early recognition of certain changes that take place in the structure of the spine, deformity and chronic pain can be prevented. I must exclude from the discussion the various senile pathological conditions of the spine, also the scolioses, and that crippling disease known as "progressive ankylosing spondylitis deformans," which starts towards the end of the growing age.

The spine is one of the most important parts of the human framework, and a study of its functions and of the disorders to which it is subject is quite fascinating. Studies of its morbid anatomy have not been so extensive in this country as on the continent, where some workers, particularly Schmorl[1] of Dresden, have had the opportunity of removing at post-mortem and examining many thousand spines from subjects of all ages, a practice which our post-mortem rules will not allow as a routine. Their researches during recent years have thrown a good deal of light on some hitherto obscure conditions, and those of Schmorl[1] have drawn particular attention to the intervertebral discs. Although there is still some controversy about some of his conclusions, his enormous experience from the examination of about 7,000 spines and his reputation as a pathologist entitle his views to a large measure of respect. Some of you will be already familiar with his work, but the subject is of considerable clinical importance, and of interest to the general practitioner as well as to the orthopædist.

Periodically one has referred to one for X-ray examination young adults complaining of a certain amount of pain in the back, often associated with a minor degree of kyphosis, and they are naturally suspected of early tuberculous disease of the spine. One finds that the radiographs may show irregular contours of the upper and lower margins of the vertebral bodies, generally in the lower dorsal region, several vertebræ being affected, and it has been often considered that these appearances were either normal variations in ossification, or a form of so-called osteo-arthritis or epiphysitis, or even the early stages of tuberculous disease.

Schmorl[1] has shown, by post-mortem studies of some of these cases who have died from other causes, that these irregular contours are due primarily to changes in the intervertebral discs, and in order to understand them I would ask you to consider for a few moments the anatomy of the normal disc and its surroundings. ( Fig. 1.)

Each disc consists of a very tough but elastic fibro-cartilaginous annular portion containing, roughly in its centre, the nucleus pulposus. The latter is a semi-fluid oval mass, a remnant of the notochord, and consists of loose fibrous tissue enclosing in its mesh-work a fluid element and a few cartilage and other cells. Normally the fluid is under considerable pressure, and the whole mass is capable of altering its position to some extent in the intervertebral space under the influence of various stresses and strains. It is separated from the bone of the vertebral body by a thin but normally highly resistant plate of hyaline cartilage. It is in this plate of cartilage that ossification occurs during the growing age, allowing for increase in the vertical length of the individual vertebral bodies.

FIG. 1.
Anatomy of Intervertebral Discs.
(Diagrammatic.)

The fibrous annular portion of the disc is firmly attached to the ligaments surrounding the spinal column and to the epiphyseal end-plates of the vertebral bodies, of which the plate of hyaline cartilage appears to form an integral part. The raised edges of the end-plates (E in diagram) are of compact bone, and are moulded in such a way as to prevent the disc as a whole from lateral, forward or backward dislocation. These epiphyseal end-plates fuse with the body of the vertebræ at ages varying between 16 and 25 years.

*The method of nourishment of the discs* is rather important. In fœtal and early infant life there are minute blood-vessels which supply the discs from the spongiosa of the vertebral body. These are mostly obliterated by the age of 8 months, and replaced by cartilage and fibrous tissue, leaving minute scars in the epiphyseal plates which may persist until adult life and be a source of weakness in the end-plate in the form of minute flaws in the cartilage. From infant life onwards the end-plates show more and more minute perforations which allow nourishing tissue-fluids to reach the discs by diffusion. The disc as a whole has been compared with "a cartilaginous sandwich, the wafers of which are the epiphyseal end-plates, and in the middle of the sandwich is the nucleus pulposus under pressure." (Mooney.)[7]

*Function.*—The functions of the discs are to give flexibility to the spine as a whole, and also to act as a series of shock-absorbers. The nucleus pulposus is mainly concerned with the latter function, and appears to have been designed not only to distribute strains and stresses evenly on the end-plates, but to act to some extent as a sort of roller-bearing on which movements of the spine can take place. If for any reason, degenerative, traumatic or otherwise, a fault develops in the cartilaginous end-plates, then the fluid elastic nucleus, being under pressure, tends to seep through the damaged spot and causes pressure-erosion of the relatively vulnerable spongiosa of the vertebral body, and the elasticity of the disc is gradually lost. The condition can be accurately compared with that of a punctured tyre. Occasionally a fault in the posterior common ligament will allow the nucleus to prolapse into the spinal canal, but this is a rare phenomenon. There is a great deal of controversy as to the exact nature and cause of these faults or fissures in the epiphyseal plates, particularly as to whether they are due to premature degenerative changes, or to traumatic influences, or to a certain mixture of both, or again whether any infective or toxic factor is at work in these cases. I cannot go into all the arguments for and against all these causal factors, but would like to mention the following considerations.

"The spine is the first organ in the body to undergo the degenerative changes of age. In this respect it is not surpassed by the intima of the blood-vessels. The cause of this must lie probably in the unique functional relations of the spine. It should be reflected what a far-reaching change of function this organ has undergone in the assumption by man of the upright habit." (Beadle.)[4]

The pressures and tensions to which the human spine is subject find no place and are not specially catered for in the spines of animals. It is interesting to note that the cartilage end-plates in the spine have to combine in one structure the function of a matrix for bony growth of the vertebræ with that of an articular bearing-surface. In a long bone, *e.g.* the lower end of the femur, these two functions are separately performed by the epiphyseal cartilage and the articular cartilage.

"The cartilage plate, then, is a most vital part of the normal disc, disturbances of whose integrity may have serious results for the whole spine." The normal resistance of the cartilage to all sorts of injurious influences is accordingly very strong, and the healthy cartilage-plate can be likened to a first-class outer tyre cover.

*Severe trauma* may, of course, rupture the plate along with the vertebral body.

*Destructive disease* starting in the bone may lead to partial or complete dissolution of the cartilage-plate (Fig. 2), but it is striking how great a resistance is offered even to severe inflammation or malignant disease originating in the vertebral body. It is probable that in the type of case I wish to discuss the development of faults in the end-plates is the result of the action not of coarse and violent trauma (although such may be the last straw which breaks the camel's back), but of the ever-active injurious influences of wear and tear working upon cartilages which are in some way inferior in their resistive power. If, for instance, the vertebral spongiosa during growth suffers any degree of osteoporosis from lack of calcium or other cause, then it will tend to be depressed by the pressure of the nucleus pulposus and the cartilage-plate will be stretched, so that it is thinner than usual at the site of the nucleus. Or again, small hæmorrhages may occur with temporary deprivation or restriction of nourishment, which may possibly start the formation of a minute fissure in the region of the bearing-surface of the end-plate.

Whatever the cause, there is no doubt that these faults occur, and owing to the "turgor"* of the fluid nucleus and the continued pressure of the weight of the upper part of the body transmitted by the spine, the nucleus begins to prolapse through the fissure. (Fig. 3.) Its contact with the bony spongiosa tends

* This post-classical and uncommon word is used by Schmorl to describe the condition of pressure of the fluid substance in the nucleus pulposus, with its tendency to swell outwards if its containing envelope is weakened or destroyed at any point. This meaning is not given in the *New English Dictionary*.

PLATE III.

Fig. 2.
Destruction of an intervertebral disc by typhoid.

FIG. 3.

Typical nucleus prolapse, magnified. Note displacement of two torn cartilage edges, the passage of a strand of nucleus tissue through the gap, and the replacement of these fibres in the narrow spaces by a nodule of newly-formed cartilage.

FIG. 4.

Slightly different example of the same. The nucleus tissue has pushed its way between bone and cartilage-plate, raising the latter. There is no new cartilage formation, but instead, a marked proliferation of bone under the prolapse, forming a barrier to further progress into the spongiosa.

### PLATE V.

FIG. 5.
Radiograph of normal dorsal vertebra at 15 years.
(Note ununited epiphyseal plate.)

FIG. 6.
Radiograph of normal adult dorsal vertebra, aged 25 years.

FIG. 7.
Case 1. Miss O. (See text.)

### PLATE VI.

FIG. 8 (*a*).

FIG. 8 (*b*).

Case 3. Miss W. (See text.)

FIG. 9.

Case 4. Miss S., at 13 years.
Scoliosis with several small nuclear prolapses.

FIG. 10.

Case 4. Miss S., at 15 years.
(Note increase in degree of scoliosis and in size of nuclear prolapses.)

to set up a defensive reaction in the latter, and a zone of sclerosis forms around the prolapsing nucleus. (Fig. 4.) If the condition can be recognized at this stage, then enforced rest will allow repair to take place before " the tyre is flat," so to speak. If not, the spine in the region of the disease will not be able to stand up to the stresses and strains to which it is constantly subjected, a disordered growth will result during the growing period, and a kyphosis, with a certain amount of wedging of the vertebral bodies, will take place.

The stage at which pain begins to be experienced is difficult to determine, but it is probable that it is not really complained of until a certain degree of kyphosis is already produced, the latter throwing abnormal strains upon the ligaments and muscles of the back.

It was Scheuermann[6] of Copenhagen who in 1920 drew attention to the irregular outlines of the ends of the vertebral shadows in radiographs in some cases of adolescent kyphosis, and he considered that these were due to some morbid process in the growth-lines between the vertebral epiphyses and the vertebral bodies. He named the disease *Osteochondritis deformans juvenilis dorsi*, and many have considered that it was an infective process affecting several vertebræ, and others have linked the condition up with the Perthe-Schlatter-Kohler group. (It is beyond the scope of this paper to discuss the relation of the vertebral condition with the other group I have mentioned.)

I showed radiographs of a case corresponding exactly with Scheuermann's description a few years ago at one of this Society's meetings.

It remained for Schmorl[1] in 1930, having made post-mortem examinations of such spines, to describe the appearances in the epiphyseal discs, with nuclear prolapses, which occur in these cases, and he considers that disc prolapses due to faults in the cartilage end-plates are most probably the primary changes, and disorders of growth resulting from alterations in the mechanical distribution of pressures and tensions once the "spring-buffer" had given way are secondary.

*Cases.*—I will now give you brief notes of a few representative cases out of the ten or so that I have had referred to me. I will first show you two skiagrams of normal dorsal vertebræ at the ages of 15 and 25 years. (Figs. 5 and 6.)

**Case 1.**—Miss O., age 15. Seen on 28th January, 1930, with kyphosis of dorsal spine, associated with some muscular weakness and pain in the lower dorsal region. Suspected of a tuberculous lesion. Condition found as shown in Fig. 7.

The bodies of the dorsal vertebræ 7–10 show marked irregularity of their upper and lower borders, with wedging, and also narrowing of the intervertebral spaces. This is an advanced stage of the disease, and corresponds exactly with the appearances described by Scheuermann.

She was treated by a spinal corset, massage, and later on exercises. Her doctor reported two years later that her carriage was very much improved, and she was generally stronger and more developed.

**Case 2.**—Miss R., aged 23. Seen on 18th January, 1932, on account of kyphosis and some pain of gradual onset. Radiographs show a similar condition affecting three or four of the lower dorsal vertebræ. Treated by rest and later by massage: the condition has improved.

**Case 3.**—Miss W., aged 28 (school teacher). Seen on 31st March, 1932. Complained of tenderness and pain in mid-dorsal region, with aching when she bent or had been standing for long, for about three months. "The back was always inclined to be weak." She had some pain there when she was 19, and also remembered a fall from a horse when she was 15. The X-ray appearances are shown in Figs. 8(*a*) and 8(*b*).

Dorsal vertebræ 6, 7 and 8 are involved, with wedging of

### PLATE VII.

(*Left.*) (*Right.*)

Fig. 11.

Case 4. Miss S. Magnified to show increase in nuclear prolapses. (Retouched.)

Fig. 12.

Case 5. Mrs. M. Calcification in nucleus pulposus and one or two small nuclear prolapses.

the bodies and some irregularity of the intervertebral spaces. The condition in this case probably started when she was in her teens, and was of slow development. Although she is now passed the growing age, her occupation as a school teacher, which involves playing games with her pupils and long hours of standing, is throwing undue strain upon an already damaged spine. I saw her a year later and the local condition was unchanged, and she still gets pain in the back, only relieved by rest.

**Case 4.** Miss S., aged 13. First seen on 11th May, 1932. (Referred to me by Dr. Powell of Weston.) This is a very interesting case, as it is complicated by the presence of a scoliosis which is said to have originated from an attack of what may have been anterior poliomyelitis when a child. When first seen she had definite scoliosis and complained of some pain in the back. The continued pain led to a suspicion of bone disease. X-ray examination (see Fig. 9) showed a moderate degree of scoliosis and slight irregularity of the intervertebral spaces in the lower dorsal region. Some small nuclear prolapses are also present (A and B), but these were not recognized as such at the time, as it was not until just after this date that I became familiar with Schmorl's work on the subject.

She was sent to a well-known orthopædic specialist in London, who, I suspect, did not realize the significance of the X-ray changes either, for he prescribed massage and exercises, and allowed her to ride and to play hockey. I saw her again nearly two years later, at the end of last year, and it will be seen from the radiograph (Fig. 10) that the degree of scoliosis has considerably increased, and that the nuclear prolapses are now quite obvious, having invaded the bodies of the vertebræ much more deeply than when previously seen. (See Fig. 11 for a comparison of the changes in two years.)

I feel that the London consultant and I made a serious mistake, due to lack of knowledge, in failing to recognize the presence of early nuclear prolapses in this girl's spine at the first examination, for this failure resulted in the omission of that rest and fixation in treatment which is essential for a cure.

**Case 5.** Mrs. M., aged about 60. Finally, a case in an older patient complaining of pain in the back, X-ray examination of whom showed degenerative changes in some of the inter-vertebral discs in the lower dorsal spine, consisting of calcification in the nucleus and one or two small nuclear prolapses. (See Fig. 12.)

Strangely enough, all the cases I have seen have been in females, and several of them school teachers, although Scheuermann[6] in his original paper stated that the male sex was affected about four times as often as the female. This may be accounted for by the fact that he examined many agricultural workers and mechanic apprentices, in whom the condition is said to be commonest.

### SUMMARY.

1. The structure and function of the intervertebral discs are described.

2. Attention is directed to their importance in maintaining the integrity of the spinal column as a highly specialized functional unit.

3. The ætiology and pathology of certain disorders of the discs occurring in young adults is discussed, with particular reference to flaws in the epiphyseal end-plates and to nuclear prolapses.

4. Five representative cases are presented, with radiographs.

5. Early recognition of the condition is regarded as important in order that the necessary rest and fixation may be given to prevent deformities, pain and "weak-back" in adult and later life.

(The thanks of the author are due to the Controller of His Majesty's Stationary Office for permission to reproduce Figs. 3 and 4.)

### REFERENCES.

[1] Schmorl. Quoted by Beadle (4).

[2] Calve and Galland, *Jour. of Bone and Joint Surgery*, 1930, xii. 555.

[3] Snoke, *Jour. of Bone and Joint Surgery*, 1933, xv. 963.

[4] Beadle, Ormond A., "The Intervertebral Discs," *Medical Research Council Special Report*, No. 161, 1931.

[5] Köhler, A., *Röntgenology*, London, 1928, pp. 208–210.

[6] Scheuermann, *Ztschr. f. orth. Chir.*, 1921, xli.

[7] Mooney, A. Craig, "Hernia of the Nucleus Pulposus," Case Report, *Brit. Jour. of Radiology*, 1934, viii. 73.

## Figures and Tables

**Fig. 1. f1:**
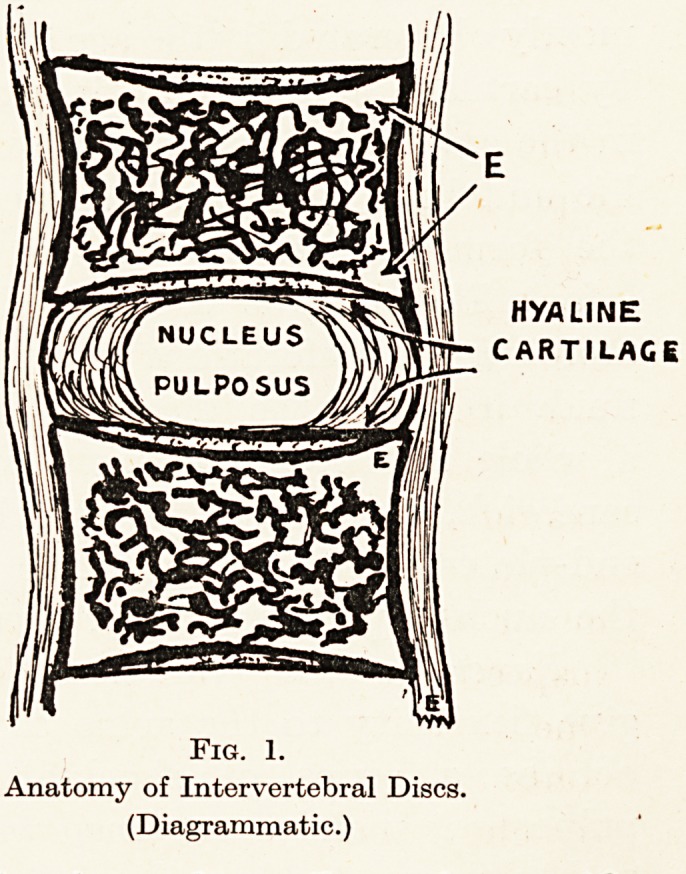


**Fig. 2. f2:**
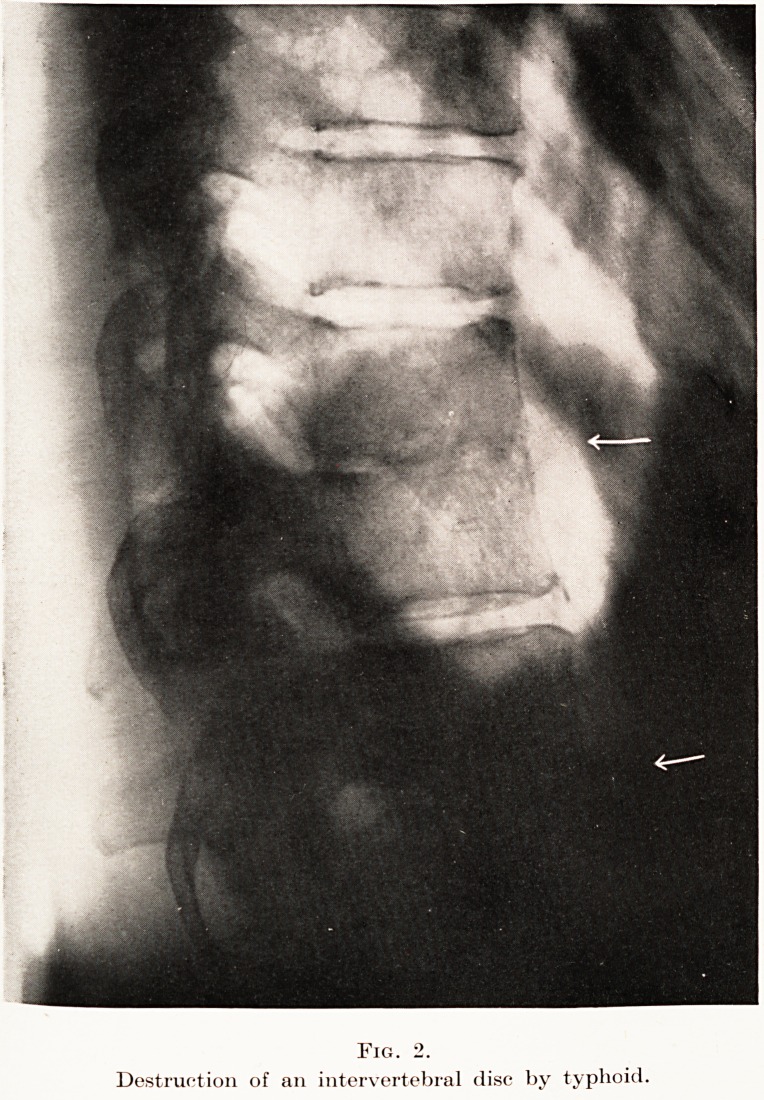


**Fig. 3. f3:**
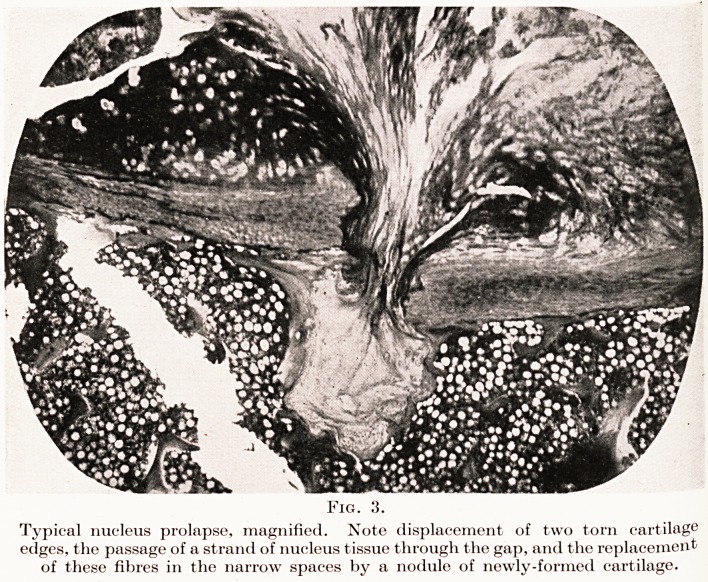


**Fig. 4. f4:**
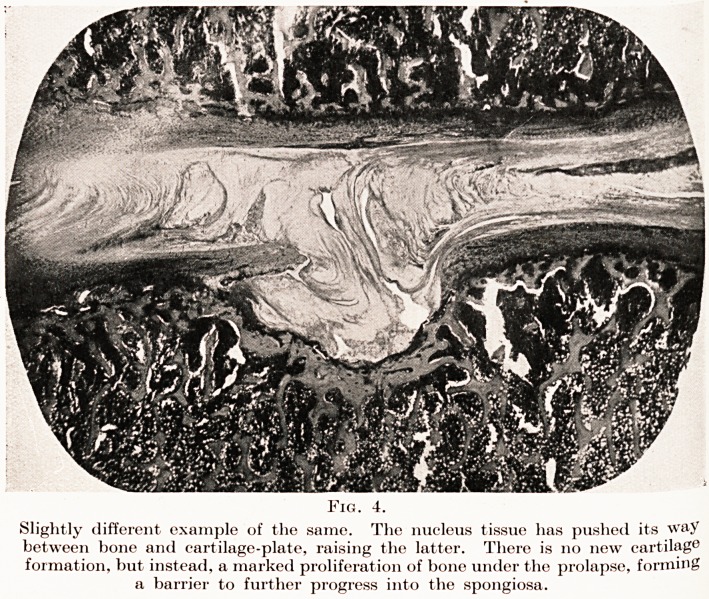


**Fig. 5. f5:**
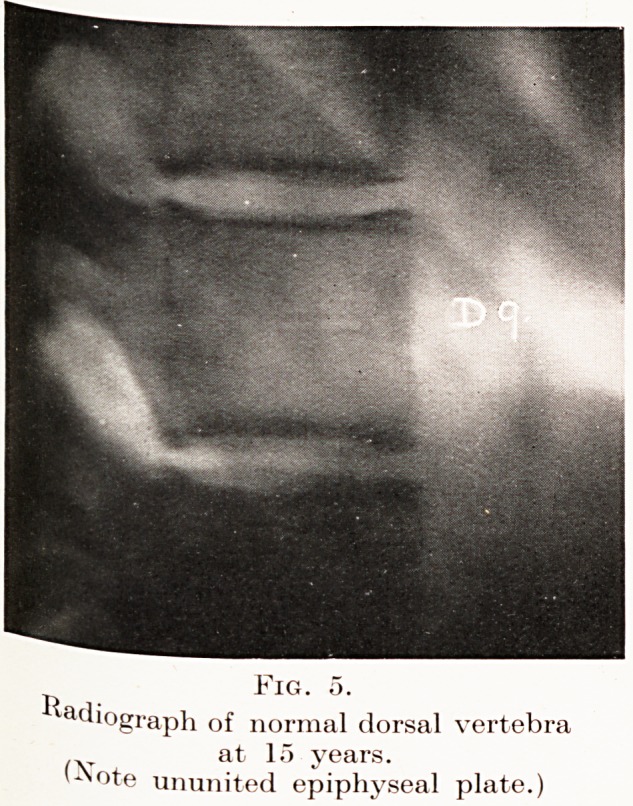


**Fig. 6. f6:**
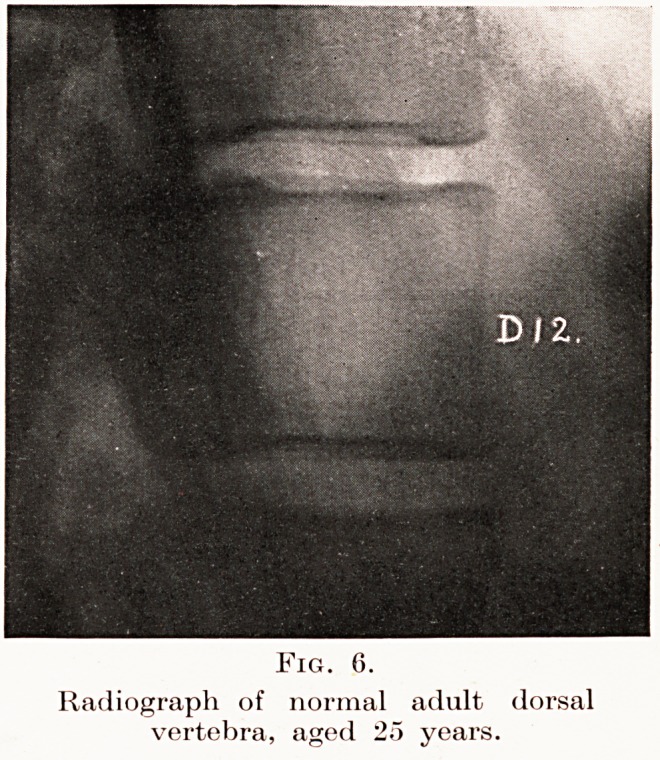


**Fig. 7. f7:**
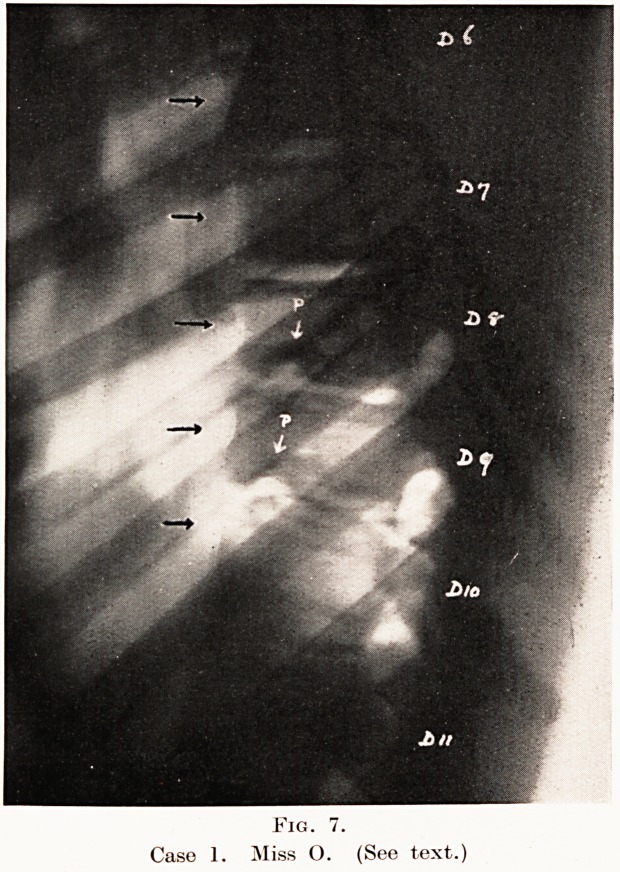


**Fig. 8 (a). Fig. 8 (b). f8:**
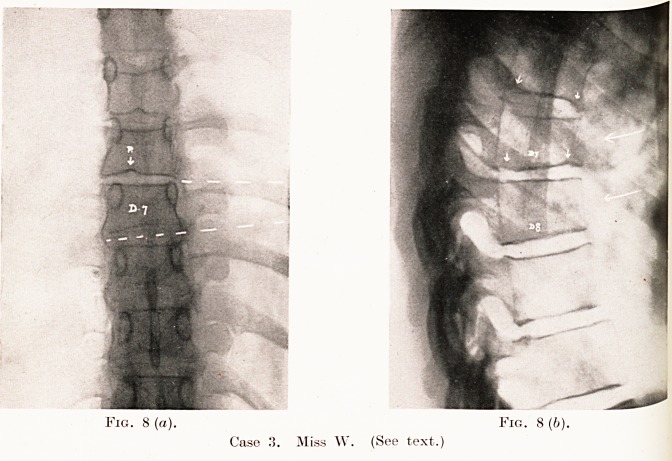


**Fig. 9. f9:**
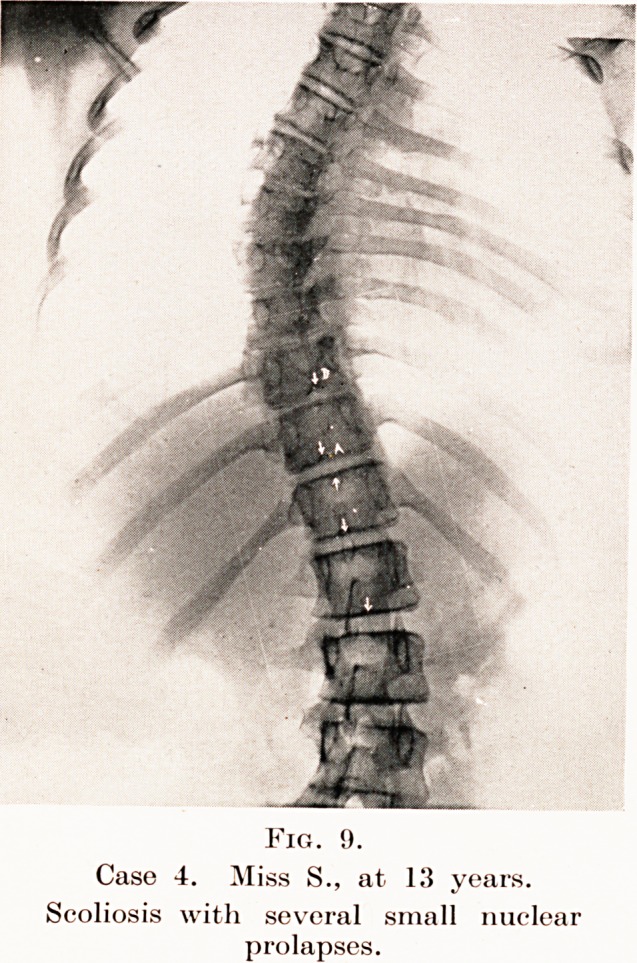


**Fig. 10. f10:**
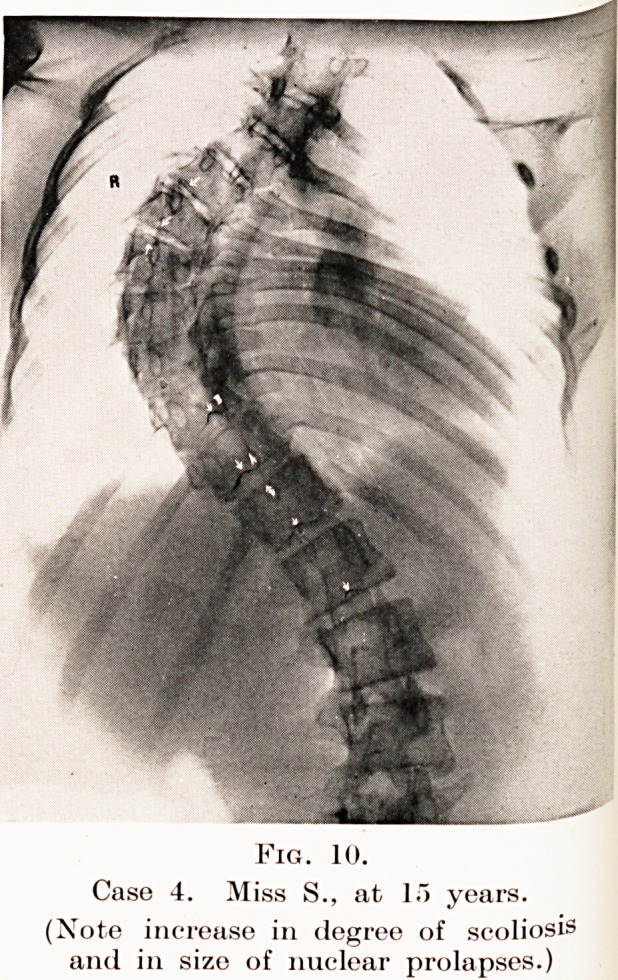


**Fig. 11. f11:**
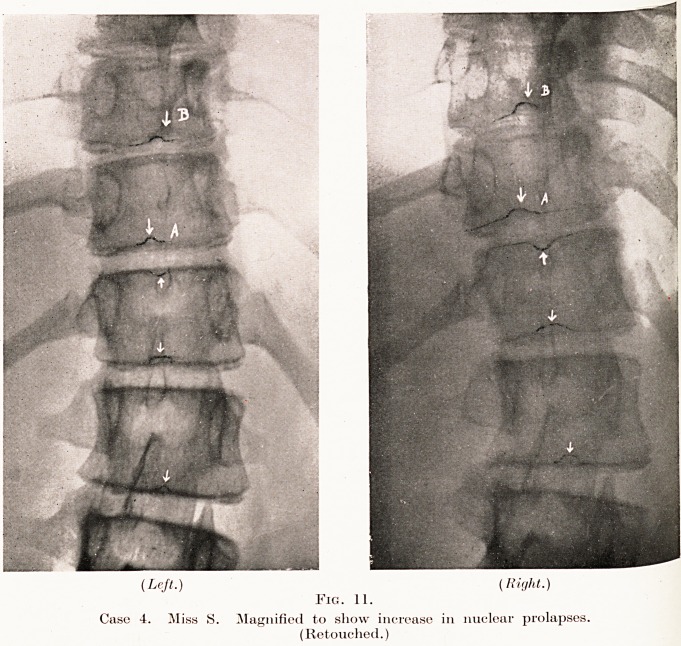


**Fig. 12. f12:**